# Feasibility of using two-dimensional axial computed tomography in pretreatment decision making for patients with early gastric cancer

**DOI:** 10.1097/MD.0000000000018928

**Published:** 2020-01-24

**Authors:** Duk Ki Kim, Sun Hyung Kang, Ju Seok Kim, Woo Sun Rou, Jong Seok Joo, Myung Hee Kim, Hyuk Soo Eun, Hee Seok Moon, Eaum Seok Lee, Seok Hyun Kim, Jae Kyu Sung, Byung Seok Lee, Hyun Yong Jeong

**Affiliations:** Division of gastroenterology and Hepatology, Department of Internal Medicine, Chungnam National University School of Medicine, Republic of Korea.

**Keywords:** computed tomgraphy, early gastric cancer, lymph node metastasis, submucosal invasion

## Abstract

Computed tomography (CT) is widely used in the pretreatment period of early gastric cancer (EGC). Only few studies have reported low accuracy of CT imaging for T and N staging in patients with EGC. However, owing to the limited number of studies, the value of CT imaging for EGC staging is not well known. Thus, we conducted a retrospective cross-sectional study regarding the associations among submucosal invasion, lymph node metastasis, and CT findings.

The medical records of patients with EGC who had surgery or endoscopic resection were reviewed in a single center from January 2011 to December 2016. We evaluated the histological type, invasion depth, and lymph node (LN) metastasis on the basis of two-dimensional CT findings.

We enrolled 1544 patients. Submucosal (SM) invasion was related to tumor size, histological type, and wall thickening or enhancement on CT images. Deep SM invasion (>500 μm) was also related to tumor size, poorly differentiated type, and abnormal CT findings (wall thickening, enhancement, and central depression). Among the patients with LN reactive positivity (0.5–1 cm), those who were female and had a tumor invasion of >1000 μm showed a higher prevalence of LN metastasis. The false-negative LN group had a higher prevalence of large tumors (>3 cm), poor differentiation, and SM invasion than the true-negative group.

Wall thickening, enhancement, and central depression on CT images might be related to SM invasion. Patients with any positive CT findings needs more attention when performing ESD

## 1 Introduction

Mucosal cancers (M cancer) <3 cm in size without ulcerations rarely show lymph node (LN) metastasis,^[1]^ so endoscopic resection (especially endoscopic submucosal dissection [ESD]) has become the main treatment method for early gastric M cancer. However, the possibility of LN metastasis in M cancer remains, and LN dissection cannot be performed during ESD. As deep submucosal (SM) and lymphatic invasions are known risk factors of LN metastasis,^[2]^ determining the lesion depth and detection of LN metastasis are vital steps for preoperative evaluation of early gastric cancer (EGC).

Two widely used preoperative methods for detecting LN metastasis and measuring lesion depth are endoscopic ultrasonography (EUS) and computed tomography (CT). Meta-analysis of EUS images revealed 87% sensitivity and 75% specificity in distinguishing T1a (M cancer) from T1b (SM cancer) cancers.^[3]^ In nodal (N) staging, EUS also revealed a remarkable outcome of 83% sensitivity and 67% specificity.^[3]^ However, EUS is an invasive, time-consuming, and operator-dependent procedure. In cases of stenosis, EUS cannot be performed. A Korean study revealed low sensitivity of EUS in T staging as compared with that of conventional endoscopy.^[4]^

Unlike EUS, CT is a routine evaluation procedure for EGC in Korea. The national health insurance system of Korea recommends preoperative CT for all patients with EGC, and considers CT performance rate as an indicator of appropriate management. The overall accuracy of CT for T staging in gastric cancer ranges from 69% to 85%,^[5–8]^ but the detection rate is significantly lower in patients with EGC, ranging from 20% to 53%.^[5,9]^ A Korean study suggested that invisibility on CT scan indicates a superficial invasive cancer,^[10]^ and many radiologists consider the absence of abnormal findings to be indicative of T1 cancer^[11]^ in spite of an insufficient level of evidence. Results of N staging are more disappointing than those of T staging, as the accuracy ranges from only 51% to 76%.^[5–7]^ As no reliable CT criteria have been established for positive LN metastasis, researchers have no choice but to use arbitrary criteria.^[11]^ CT gastrography showed a more reasonable and higher detection rate of EGC,^[12]^ but three-dimensional (3-D) reconstruction is as time-consuming as EUS.

For endoscopists, cancer lesions can be easily found with conventional upper endoscopy, and several endoscopic diagnostic criteria for prediction of tumor invasion depth in EGC have been developed.^[13,14]^ After the discovery of EGC, the major concern of endoscopists is the treatment strategy decision, whether the lesion is an indication for endoscopic or surgical resection. CT may play an important role if CT findings can provide valuable information regarding SM invasion or the true positivity of LN metastasis. Especially in the expanded indications for ESD, endoscopists are constantly concerned of latent or false-negative LN metastasis during preoperative evaluation. However, only few studies focused on CT findings related to SM invasion or true-positive LN metastasis. Thus, we conducted a retrospective cross-sectional study on the association between pathological data such as SM invasion, LN metastasis, lymphovascular invasion (LVI), histological type, and two-dimensional (2-D) CT findings.

## 2 Materials and methods

### 2.1 Study population

The medical records of patients with EGC who had surgery or ESD between March 2011 and March 2016 in a tertiary hospital in Daejeon, South Korea, were reviewed. Patients without preoperative CT, with poor imaging profiles (CT scans taken in other hospitals), with a history of other malignancy within 5 years, and with difficulties in the evaluation of LN status due to infection or perforation were excluded. Patients who underwent virtual CT gastroscopy with 3-D reconstruction were also not included.

### 2.2 CT examination

CT was performed using a 64-slice MDCT system (Siemens Medical Systems, Erlangen, Germany) and dual-layer detector spectral CT scanner (Philips Healthcare, Amsterdam, the Netherlands). All the patients received 500 ml of water as an oral contrast agent approximately 15 minutes before the examination and an additional 500 ml immediately prior to the study.

In all the patients, a total volume of 3 ml/kg nonionic contrast material containing iobitridol 767.8 mg/ml (Xenetix 350 injection; Geurbet, Korea) was administered intravenously at a rate of 2.5 ml/second using an automated injector device through an 18-G intravenous catheter located in the antecubital vein. Imaging was started within 2 minutes after administration of the contrast agent.

A MDCT system was used, with the following parameters: detector collimation, 0.6 mm; table speed and rotation, 0.5 mm; table pitch, 0.6; effective section thickness, 3 mm; reconstruction intervals, 3 mm, 120 kVp; and 230 mAS (Auto). Dual-layer detector spectral CT scans were performed with (a) a slice thickness of 1 mm (reconstructed slice thickness of 5 mm), (b) pitch of 0.359, (c) collimation of 64 × 0.625, (d) increment of 0.5 mm (reconstructed increment of 5 mm), (e) rotation time of 0.33 seconds, and (f) 320-mA tube current time product and 120-kVp tube voltage.

### 2.3 Image analysis

**Figure 1:**
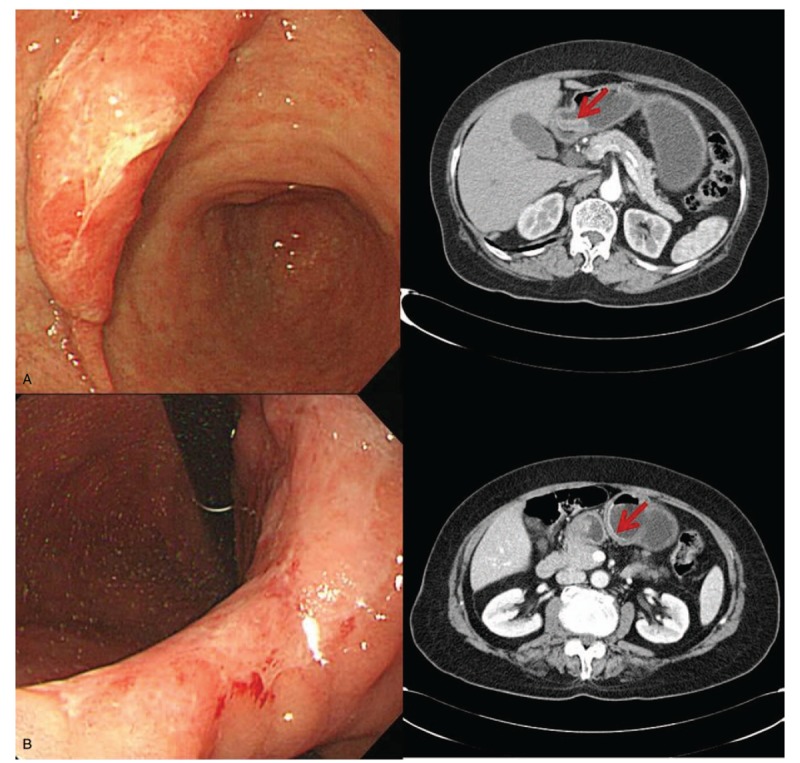
(A) 77-years-old-female patient. EGC with SM invasion was found as an elevated lesion in gastroscopy and wall thickening in CT scan. (B) 78-years-old-female patient. She had also EGC with SM invasion and shallow depressed lesion was found in gastroscopy. In CT scan, wall enhancement was noted.

**Figure 2:**
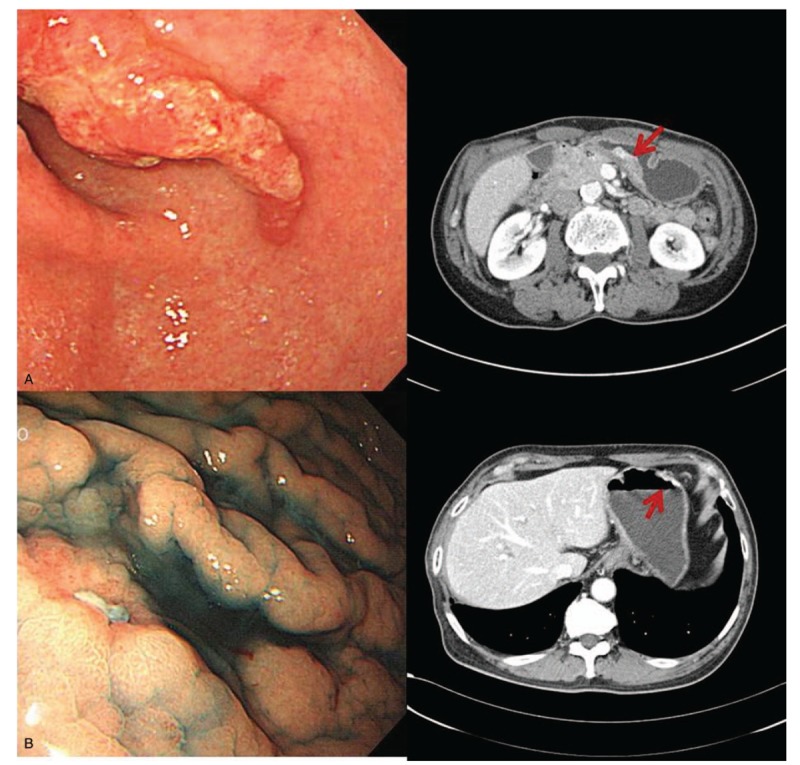
(A) 71-years-old-male patient. He had EGC with deep SM invasion at antrum. Elevated lesion was found in gastroscopy and polypoid lesion was also found in CT scan. (B) 68-years-old -male patient. Central depressed lesion was found in gastroscopy and CT scan.

Three experienced abdominal radiologists independently reviewed the CT images. They had full access to clinical information via the electronic medical record system such as the tumor location detected with endoscopy, symptoms of the patients, and histological type of cancer. On the basis of the medical records, findings from CT readings were classified on the basis of 5 criteria as follows: no visible lesion, wall thickening, wall enhancement, polypoid lesion, and central wall depression (Figs. 1 and 2). LN enlargement of >10 mm was defined as metastatic lymphadenopathy. Perigastric LN with a short axis of >6 mm and extraperigastric LN with a short axis of >8 mm were also considered metastatic lymphadenopathies. A LN size of <5 mm was regarded nonsignificant. LNs of >5 mm in size but do not fulfill the criteria for pathologic lymphadenopathy were defined as reactive lymphadenopathy.

### 2.4 Pathological examination

A single, experienced pathologist reviewed all surgical and ESD specimens on the basis of the *World Health Organization Guidelines, Fourth Edition*.^[15]^ The histological types of EGC were classified as well to moderately differentiated, poorly differentiated, pure signet ring cell carcinoma (SRC), poorly differentiated types with SRC components, mixed types, medullary carcinomas, and poorly cohesive carcinomas other than SRCs. The histological type was determined according to the dominant cell type (>50%). SRC and poorly cohesive carcinoma other than SRC were differentiated because pure SRC has a more favorable prognosis than other types of poorly cohesive carcinoma.^[16,17]^ Surgical treatment was performed with subtotal or total gastrectomy with D1+α or D2 lymphadenectomy. LN analysis was omitted for the ESD specimens, and only analysis for LVI was performed. Pathological specimens obtained after gastrectomy were sectioned at 4-mm intervals, and those obtained after ESD were sectioned at 3-mm intervals.

### 2.5 Statistical methods

Statistical analysis was performed using the SPSS version 18.0 software (IBM Corporation, Chicago, IL, U.S.). Logistic regression analysis was used for assessment of the relative risks of SM invasion, LVI, and false-negative LN metastasis. The accepted level of statistical significance was *P* < .05.

### 2.6 Ethics statement

This study was approved by the institutional review board of Chungnam National University Hospital (IRB No. 2018-08-028), and written consent was waived because of the retrospective design of the study. This study was conducted in accordance with the principles of the Declaration of Helsinki and International Conference for Harmonization.

## 3 Results

The study consisted of 1544 patients, with a mean age of 63.3 years, of whom 1083 were male and 760 (49.2%) received ESD. Tumor size, depth of invasion, LVI, and frequency of CT findings differed depending on the treatment modality used (Table 1). Larger tumor size, poor differentiation especially in medullary carcinoma and the mixed type; positive LVI; and any positive findings on CT scan (including lymphadenopathy) were associated with SM invasion (Chi-Squared test, *P* < .05). Tumor size, histologic type, and LVI were significant risk factors of SM invasion in logistic regression test (*P* < .05). Deep SM invasion more than 500 μm was also related to these findings. Tumor size, histologic type, CT findings, and LVI were risk factors of deep SM invasion (Chi-Squared test, *P* < .05; Table 2). Unlike SM invasion, deep SM invasion was not related to tumor size in the logistic regression analysis. Histological type and LVI were risk factors of deep SM invasion in the multivariate analysis (Table 3).

**Table 1:**
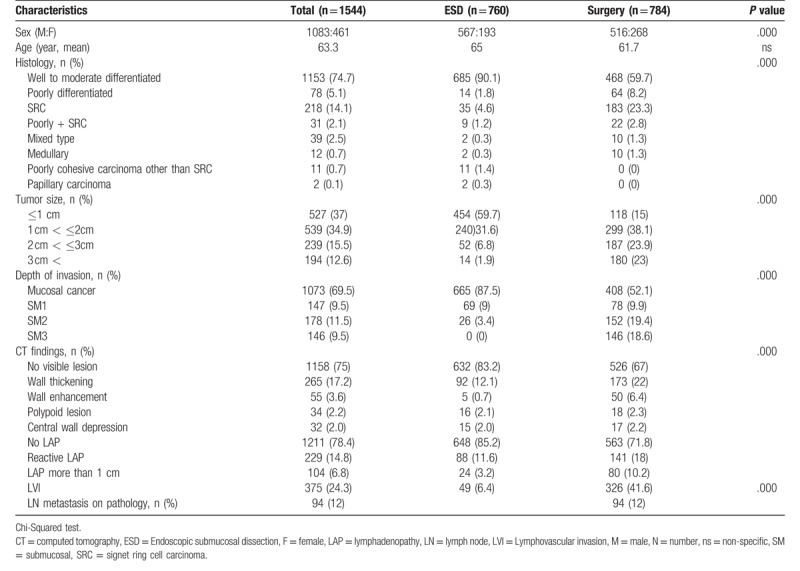
Demographic features of the study population. There are several differences depending on treatment modality.

**Table 2:**
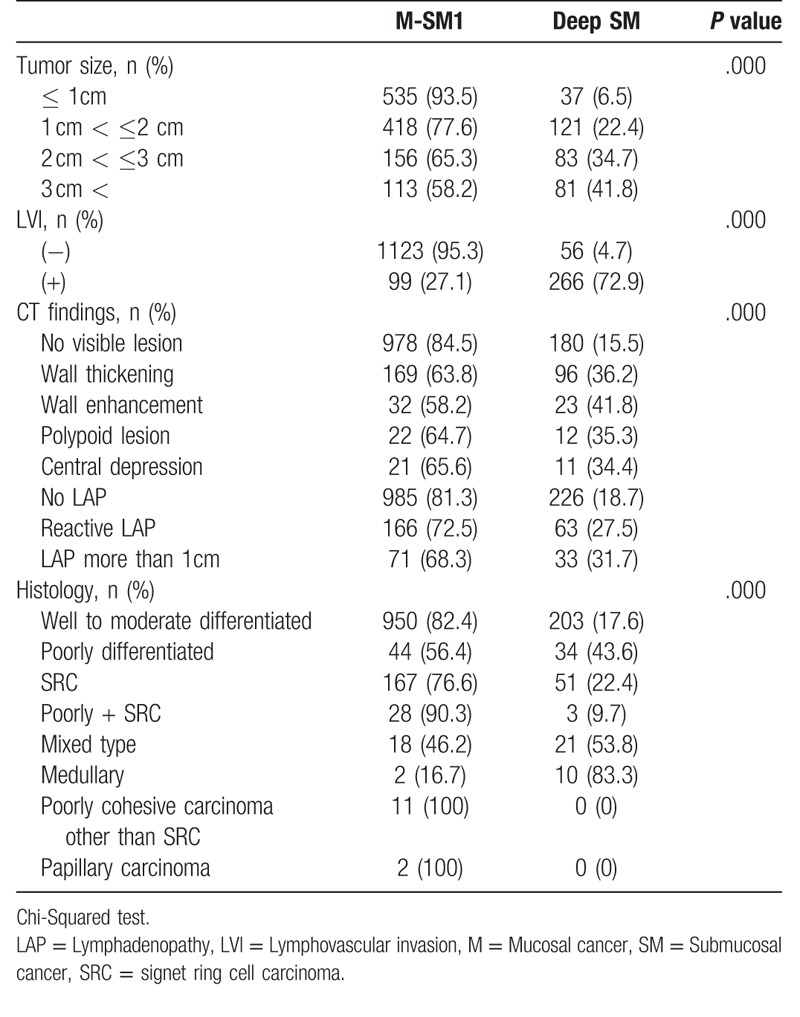
Risk factors for deep SM invasion.

**Table 3:**
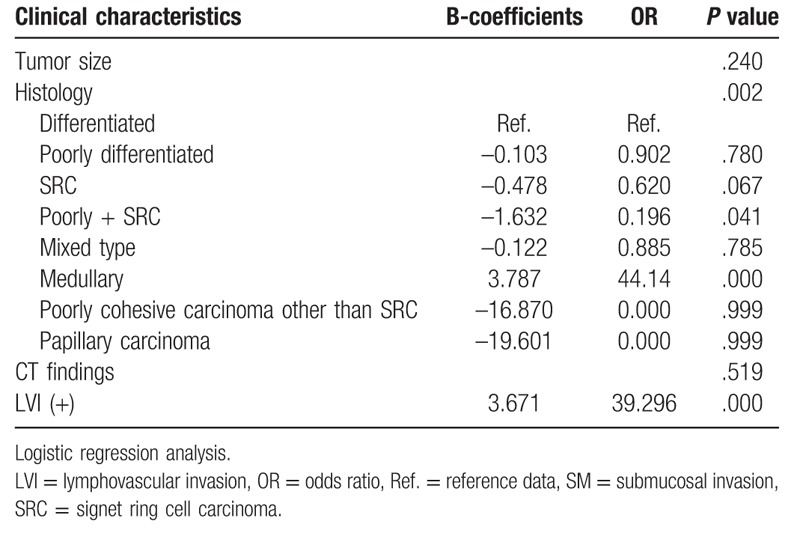
Multivariate analysis of deep SM invasion.

LN metastasis was related to invasion depth, histological type, tumor size, LVI, and positive CT findings in the operation group (Table 4). In the multivariate analysis, tumor size and LVI were the risk factors of LN metastasis (Table 5). Of the patients, 141 showed reactive LN on the preoperative CT scan, of whom 20 (14.1%) revealed LN metastasis after postoperative pathological review. The preoperative CT images of the patients with reactive LN revealed that tumor size, invasion depth, LVI, and female sex were related to real LN metastasis (Chi-Squared test, *P* < .05). In the logistic regression analysis, only female sex was a risk factor of false-negative LN in the reactive group (Table 6).

**Table 4:**
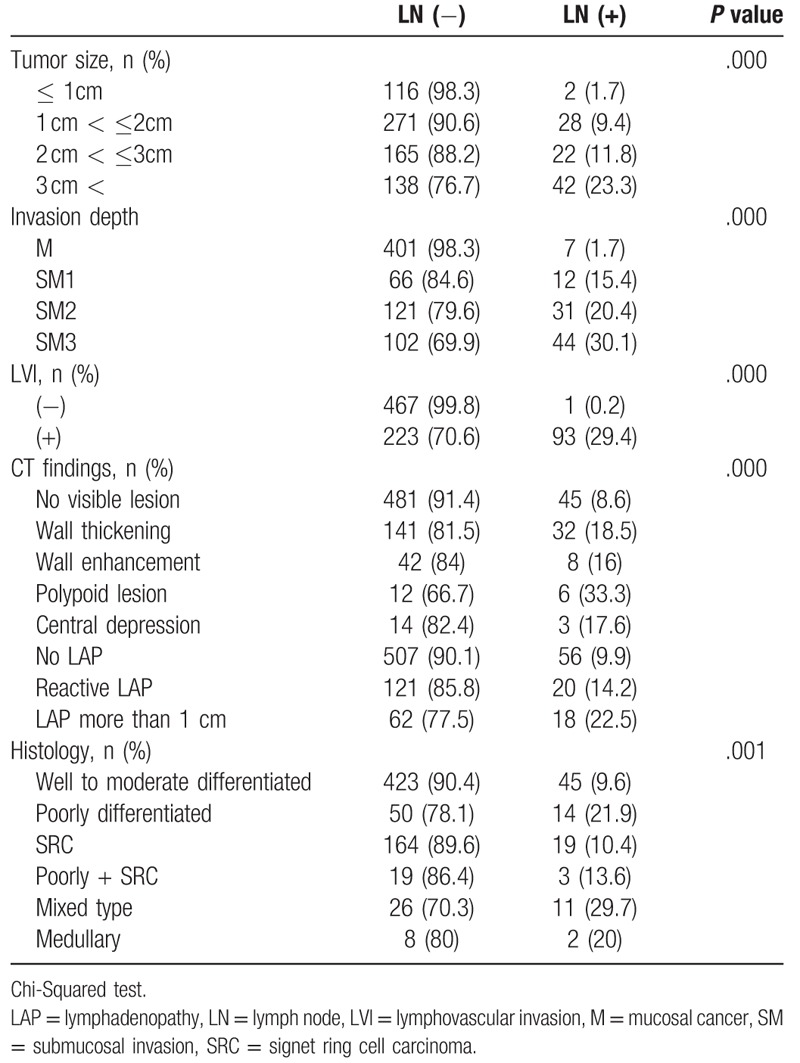
Risk factors of LN metastasis in surgical resection cases.

**Table 5:**
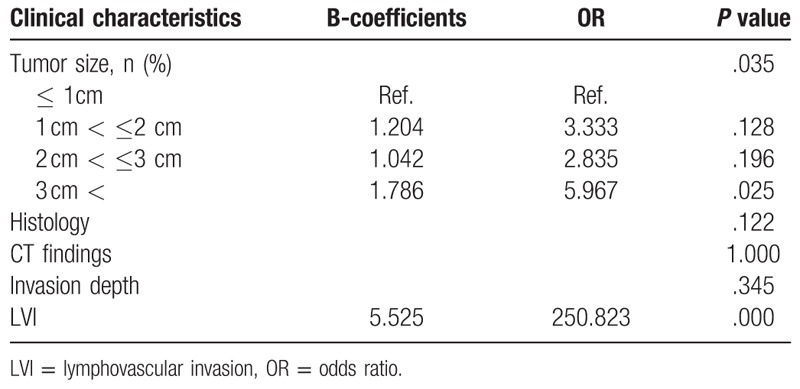
Multivariate analysis of LN metastasis in surgical cases.

**Table 6:**
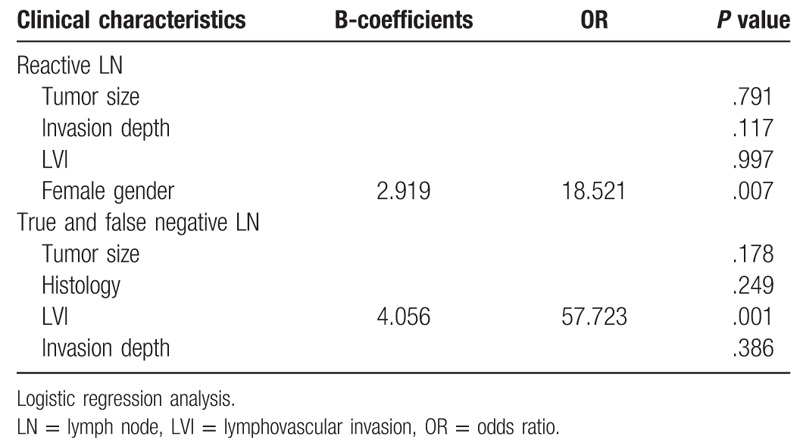
Risk of LN metastasis in preoperative negative LN patients.

We also conducted a subgroup analysis that compared the groups with true-negative and false-negative LNs on preoperative CT images. The false-negative group showed larger tumor sizes, poorer differentiation, and deeper SM invasion than the true-negative group (Chi-Squared test, *P* < .05). Positive LVI and CT findings except LN size were associated with LN metastasis (Chi-Squared test, *P* < .05). In the multivariate analysis, only LVI was a risk factor of LN metastasis (Table 6).

## 4 Discussion

In the preoperative evaluation of EGC, both CT and EUS are widely used to evaluate depth of invasion and LN metastasis. While these procedures are routinely performed, unexpected pathological findings are still reported, such as deep SM invasion or positive LVI after ESD, M cancer with positive LN metastasis after surgical resection, and latent LN or organ metastasis after several-years-ago gastrectomy or ESD. In cases beyond indication for ESD, additional surgical resection is mandatory, but patients might refuse surgical resection because of old age, comorbidity, and fear of surgery. While ESD is a safe, convenient, and effective modality for treating superficial EGC, LN dissection cannot be performed. The present study was conducted to find a correlation between CT findings and pathological results after ESD or gastrectomy. By understanding the correlation, the proper candidates for ESD could be isolated from among patients with EGC. Moreover, we identified significant CT findings for which ESD should not be performed. Radiologists have considered that the absence of positive findings indicate superficial EGC.^[10,11]^ A previous Korean study that correlated CT findings with pathological results revealed a higher detection rate for deep EGCs than for superficial EGC.^[10]^ Our results are also in line with the those of the previous study, as almost all the non-visible lesions were M cancers and positive findings correlated with SM invasion. Another Japanese study focused on the differentiation between ulcerative EGC and advanced gastric cancer (AGC).^[18]^ In this study, AGC showed more peak enhancement on CT scan than ulcerative EGC. Deeper invasion may appear as a clear and strong enhancement on CT scan. This finding was also supported by our study results. Wall thickening, wall enhancement, and central depression were findings of SM invasion in our study. Clear enhancement on CT scan increases the possibility of SM invasion. Another study insisted that axial images without 3-D reconstruction may miss small, flat, or depressed lesions.^[19]^ In this study, missed lesions on axial images were mucosal cancers or cancers located in the antrum.^[19]^ Non-visible lesions on CT scans were mostly M cancers in our study (75.2% of M cancers vs 24.8% of SM cancers, *P* < .05). Thus, the absence of CT abnormalities may be an indication for ESD, and small cancers with no CT abnormalities may also be candidates for ESD.

Although nodal staging is another important role of preoperative CT, the accuracy of nodal staging is quite disappointing. Owing to the absence of standard diagnostic criteria of LN metastasis on CT scan, many criteria have been used. Criteria using size estimation have been widely used for the diagnosis of pathologic lymphadenopathy. We also used criteria using size estimation, which have been used in other previous studies.^[20,21]^ Metastatic LN was defined as any LN >10 mm in size, perigastric LN with a short axis of >6 mm, and extraperigastric LN with a short axis of >8 mm. Criteria using size estimation have several limitations. First, it ignores the shape and enhancement pattern of LN. Second, LN enlargement may appear because of inflammation. Differentiation between pathologic and reactive LNs are challenging to physicians. In our study, tumor size, histological type (poorly differentiated and mixed type), SM invasion, positive LVI, and other positive findings on preoperative CT scans were related to postoperatively pathologically confirmed LN metastasis in patients who underwent surgical resection (Chi-Squared test, *P* < .05). However, we could not provide the clinical meaning of the preoperative CT in the logistic regression analysis.

False-negative LN was related to sex, tumor size, and deep SM invasion in the reactive LN group. Reactive or negative LN metastasis on preoperative CT scans might be a false negative when the pathological data after ESD show deep SM invasion, large tumor size, and positive LVI. Deep SM invasion of >500 μm and LVI are risk factors of LN metastasis and indications for additional surgery.^[22–24]^ Our results support previous studies, and in such situations, one must consider additional surgery after ESD despite negative findings from the preoperative CT scan. Unfortunately, we could not explain why female sex showed a high rate of LN metastasis in the reactive LN group.

Gastric adenoma, also known as gastric dysplasia, is classified into high- and low-grade dysplasias.^[25]^ Many studies reported upgrade of pathology from high-grade dysplasia to early gastric cancer after ESD of gastric dysplasia.^[26–28]^ Preoperative CT might not be performed in most cases of gastric adenoma. LN enlargement may appear on CT after ESD because of inflammation and mucosal damage. In these cases, physicians may be confused when differentiating pathologic LN from reactive change. The results of our study may aid diagnosis of pathologic lymphadenopathy. Differentiated cancer with small, mucosal, or superficial SM invasion could be observed in spite of LN enlargement on post-ESD CT scans.

Our study has several limitations. First, it is a retrospective study. Our data were based on medical records, and pathological or radiological reassessment was not performed. Image analysis and pathological review were not based on a specific, constant protocol. However, all the professionals in our institution are highly experienced. Second, the sample size may not be sufficient, and the study was conducted in a single center. Lastly, patients with virtual CT gastroscopy with 3-D reconstruction were not included. Virtual CT gastroscopy with 3-D reconstruction was performed in a few patients (36 men and 26 women) during the study period. Virtual CT gastroscopy with 3-D reconstruction has been reported to be more sensitive than conventional axial 2-D CT.^[29]^ 3-D virtual CT endoscopy might provide more detailed information about SM invasion and nodal metastasis than 2-D axial CT.

In conclusion, wall thickening, wall enhancement, and central depression on CT images are risk factors of SM invasion. Female sex, poor differentiation, deep SM invasion, and large tumor size were risk factors of false-negative LN metastasis. When assessing the absolute indication range for endoscopic submucosal dissection, the possibility of false-negative LN metastasis on CT scans might not be problematic.

## Author contributions

**Conceptualization:** Sun Hyung Kang, Jae Kyu Sung, Byung Seok Lee, Hyun Yong Jeong.

**Data curation:** Duk Ki Kim, Myung Hee Kim, Seok Hyun Kim.

**Formal analysis:** Myung Hee Kim, Seok Hyun Kim.

**Investigation:** Duk Ki Kim, Sun Hyung Kang, Ju Seok Kim, Hee Seok Moon, Jae Kyu Sung.

**Methodology:** Sun Hyung Kang, Ju Seok Kim, Hee Seok Moon, Eaum Seok Lee.

**Project administration:** Sun Hyung Kang, Jong Seok Joo, Eaum Seok Lee.

**Resources:** Woo Sun Rou, Jong Seok Joo, Hyuk Soo Eun.

**Software:** Woo Sun Rou.

**Supervision:** Sun Hyung Kang, Byung Seok Lee, Hyun Yong Jeong.

**Validation:** Hyuk Soo Eun.

**Writing – original draft:** Duk Ki Kim.

**Writing – review & editing:** Sun Hyung Kang.

Sun Hyung Kang orcid: 0000-0002-1913-4346.
